# Cross-Linguistic Influence on L2 Before and After Extreme Reduction in Input: The Case of Japanese Returnee Children

**DOI:** 10.3389/fpsyg.2020.560874

**Published:** 2020-09-18

**Authors:** Maki Kubota, Caroline Heycock, Antonella Sorace, Jason Rothman

**Affiliations:** ^1^Department of Language and Culture, UiT The Arctic University of Norway, Tromsø, Norway; ^2^School of Philosophy, Psychology & Language Sciences, The University of Edinburgh, Edinburgh, United Kingdom; ^3^Department of Language and Culture, UiT The Arctic University of Norway, Tromsø, Norway; ^4^Department of Language and Culture, Nebrija University, Hoyo de Manzanares, Spain

**Keywords:** cross-linguistic influence, genitive (possessive) relations, verb argument construction, Japanese returnees, longitudinal study

## Abstract

This study investigates the choice of genitive forms (the woman’s book vs. the book of the woman) in the English of Japanese-English bilingual returnees (i.e., children who returned from a second language dominant environment to their first language environment). The specific aim was to examine whether change in language dominance/exposure influences choice of genitive form in the bilingual children; the more general question was the extent to which observed behavior can be explained by cross linguistic influence (CLI). First, we compared the choice of genitive form between monolingual English speakers and bilinguals who had recently returned to Japan from an English speaking environment. Second, we tracked changes in genitive preference within bilingual children, comparing their performances upon return to Japan to those of one year later. Results show that CLI alone is insufficient to explain the difference in genitive evaluation between bilinguals and monolinguals, as well as the intra-group bilingual variation over time. We suggest that both CLI and general processing considerations couple together to influence the changes in genitive preference.

## Introduction

Studies examining (potential) cross-linguistic influence (CLI) provide valuable data for fully understanding how both languages of bilinguals develop and interact over time. In the present study, we define CLI as influence on behavior exhibited by bilinguals that can reasonably be attributed to their other language, that is, not otherwise explainable by developmental processes also noted in monolingual language development (be it delay or acceleration). For example, Japanese learners of English may produce phrases such as *sale’s spelling* (rather than *the spelling of “sale”*)*−*a form that is rarely observed in a developmental stage of English monolingual speakers (Tomiyama, 2000). This behavior can reasonably be attributed to CLI from L1 Japanese due to the fact that Japanese only allows pre-nominal possessive construction that linearly maps onto the English *s-*genitive form.

A large body of literature has been examining the circumstances in which CLI occurs between the two languages of a bilingual. An explanation that figures prominently in this literature is language dominance. On the one hand, studies have shown CLI to take place from the dominant language to the non-dominant language (Yip and Matthews, 2000; Argyri and Sorace, 2007). But others (Müller and Hulk, 2001; Paradis and Navarro, 2003; Sorace and Filiaci, 2006) have instead proposed that the linguistic properties of the two languages*−*namely, structural overlap and interface condition, discussed further below*−*determine the occurrence and the directionality of CLI.

The aim of the current study is two-fold. First, in Study 1, we examine the role of linguistic properties in predicting CLI in Japanese-English bilingual children by comparing them to a control group of monolingual peers. By testing the bilinguals within weeks of their return to Japan, we aim to capture their acquired competence in English after significant exposure to native English in an immersion context (mean time of immersion: 4 years). At this point any observable influence from Japanese would be especially significant, speaking to the robustness of CLI effects (i.e., taking place despite ample exposure and high quality of L2 input). Further, the performance of the bilinguals in Study 1 also serves as their own baseline in Study 2, where they are tested after a year of reintegration into Japan. Study 2 thus probes for changes over time within individual speakers, which we also hypothesize will follow a particular pattern induced by CLI effects. A unique aspect of this study is manifested in Study 2, that is, only by studying returnees, can we meaningfully see how CLI and reduction of input interact in a context of L2 attrition. This special context and our longitudinal approach permit us to investigate the effects of a shift in environmental language dominance, as it changes from second language dominant (an English-speaking environment) to first language dominant (Japan).

The studies focus on two grammatical phenomena: the choice of genitive forms and verb/argument order. Starting with the first of these, in English there are two principal ways to express a possessive relationship within a noun phrase: the pre-nominal possessive form, or *s-*genitive (e.g., *the table’s leg*), and the post-nominal possessive form, or *of-*genitive (e.g., *the leg of the table*). In Japanese, there is only a pre-nominal genitive, where the pre-nominal possessive is suffixed with the particle *no* (e.g., *Hanako no koppu*; Hanako’s cup). In terms of verb/argument order, Japanese is an SOV language whereas English is SVO. These differences lead to predictions about CLI. First, we expected CLI to occur in genitive forms but not verb/argument order (when comparing bilinguals to their monolingual counterparts), due to the fact that genitive forms meet two conditions of CLI (Müller and Hulk, 2001): structural overlap and integration of pragmatic and/or semantic factors, while verb/argument order fulfills neither of these conditions. Second, we expected the effects of CLI from L1 Japanese to L2 English to increase over time after the bilingual returnee children have returned to an L1 dominant environment with minimal L2 exposure.

### Explaining CLI

#### Language Dominance

Since many bilingual children are more proficient in or more exposed to one language than the other, some studies have argued that CLI is unidirectional, taking place from the dominant to the non-dominant language (Yip and Matthews, 2000; Paradis, 2001; Argyri and Sorace, 2007; Kupisch, 2007; Nicoladis, 2012). For example, Argyri and Sorace (2007) found that CLI in syntactic structures occurred from English to Greek among bilingual children, but this effect was found only among bilinguals who were dominant in English, not in children who were dominant in Greek. It should be noted that “dominance” is defined in a number of different ways in the literature. For example, Argyri and Sorace (2007) as well as Serratrice et al. (2009) define children’s “dominant language” as the majority language of the environment (i.e., Italian in Italy), while Yip and Matthews (2000); Nicoladis (2012) use proficiency measures such as mean length of utterance (MLU) or the Peabody Picture Vocabulary Test (PPVT) to determine the dominant language of bilingual children. It remains the case that no uniform definition exists for this term (for further discussions see Treffers-Daller and Silva-Corvalán, 2016). In this paper we follow the studies that define language dominance in terms of the relative amount of language exposure the child receives in each language.

Tomiyama (1999, 2000) is an example of a study that found evidence of L1 CLI due to reduced L2 exposure. In this study, Tomiyama tracked L2 English progress of a Japanese returnee child longitudinally over the course of 33 months. The child was 8 years old at the time of his return to Japan and data was collected once a month using free conversation and a story-telling task. In the second stage of data collection (from 20 months to 33 months), the child used erroneous *s-*genitive forms such as “^∗^the window’s place.” Tomiyama concluded that the inappropriate use of the *s-*genitive is an indicator of L1 CLI, since the genitive form in Japanese resembles the linear order in the *s-*genitive in English. Moreover, studies from Yoshitomi (2007); Snape et al. (2014) reveal that aspectual domain in L2 English showed some signs of attrition after 8−12 months of returning to Japan.

#### Linguistic Properties and Processing

An alternative hypothesis for explaining CLI*−*focusing on the internal structures of the two languages*−*was first proposed by Hulk and Müller (2000); Müller and Hulk (2001), suggesting that linguistic phenomena subject to CLI must (a) involve two modules of grammar (e.g., syntax/pragmatics) and (b) have similar structures but also be “ambiguous.” Here, ambiguity refers to cases when there is an overlap between the two languages in the sense that one language allows only one form to express a particular function, whereas the other language has two. This also determines the directionality of CLI: the language with one form influences the language with two forms.

Elaborating this idea for adult bilinguals, Sorace and Filiaci (2006) propose that structures that are conditioned by contextual or pragmatic factors are especially difficult to acquire and are also more vulnerable to effects of attrition than structures that only involve syntactic aspects of the language. It is important to note, however, that unlike Müller and Hulk, Sorace and Filiaci do not make any explicit claims about the directionality or source of CLI, but rather propose that there are different conditions on syntactic realization in bilingual acquisition, which depend to a greater or lesser extent on coordination with “external”(pragmatic, contextual) factors (Sorace, 2011, 2016). The principal empirical test bed for this hypothesis has been the distribution of pronominal forms. For example, in Italian, there are two ways to express pronominal subjects: overt and null pronouns. The choice of these two forms is governed by pragmatic factors*−*a null pronoun is used when referring to the topic of the previous sentence, whereas an overt pronoun is used to refer to a non-topical antecedent. In contrast to Italian, English only has one form, overt pronouns (e.g., *he*, *she*) to express the same functions. Thus, according to Sorace and Filiaci (2006), English-Italian bilinguals may behave differently from monolinguals in the comprehension and production of pronominal forms in Italian, not only because of the linguistic differences between Italian and English, but also because of the processing load related to linking pronouns to their antecedents in a pragmatically appropriate way in real time.

Sorace (2011, 2016), moving further away from an exclusively generative linguistic framework, elaborated on these ideas by proposing that integration of pragmatic and contextual conditions may be particularly difficult to process for the bilinguals due to the extra cognitive demands it requires. Since cognitive resources are needed to adapt to changing contextual conditions that require one pronoun or the other, bilinguals may experience more competition for cognitive resources, since they also have to inhibit the unwanted language that is not in use. Bilinguals solve this pressure by overextending the scope of the overt pronoun, which is the most explicit form and thus used as a “default” pronominal form to relieve the processing demands caused by the need to integrate pragmatic and/or contextual information. Following this, appealing to cognitive resource allocation as a source of divergence between monolinguals and bilinguals (rather than CLI from a non-null subject language) could better explain why overextension of overt pronouns is found even in bilingual adults (e.g., Lozano, 2006; Margaza and Bel, 2006) as well as bilingual children (Serratrice et al., 2009; Sorace and Serratrice, 2009) speaking two null subject languages. These studies point to the need to examine the interaction between linguistic and non-linguistic (general cognitive) factors in explaining developmental trajectories, particularly for language structures sensitive to ‘external ‘contextual conditions.

So far, we have discussed possible explanations for the apparent constrained effects of CLI patterns in bilingual children, focusing on language dominance, linguistic properties, and cognitive load. These factors, however, do not necessarily have to co-occur for CLI to take place. For instance, there is no shortage of empirical evidence showing CLI in the absence of a structural overlap between the two languages of the bilingual and/or for properties that are not clearly interface structures (see e.g., Yip and Matthews, 2000; Nicoladis, 2002; Foroodi-Nejad and Paradis, 2009; White, 2011; Rothman et al., 2019). Nevertheless, the factors of interest here clearly play a role in how CLI obtains, while it is still uncertain how they might interact to drive CLI. This study contributes, then, by offering a means to tease apart some of these factors. More specifically, it allows us to isolate, under conditions of dramatic reduction in input in one of the two languages, the relative contribution of distinct factors, e.g., overall language dominance versus the types of structures involved, as well as potential interactions.

### Genitive Variation in English

Having now discussed the essential concepts relating to CLI, we now turn to explain the choice of syntactic phenomena that we focussed on in our study, beginning with variation in genitive structures in English. There is considerable debate in the literature as to what determines the choice between the *of*-genitive and *s*-genitive, but also a degree of consensus on some principal factors. These include semantic properties such as animacy and the type of possessive relation, and discourse related factors such as topicality. Animacy is often regarded as the central factor in genitive choice. Several corpus studies (Jucker, 1993; Leech et al., 1994; Gries, 2002; Stefanowitsch, 2003; Rosenbach, 2005) have examined the relative frequency of the two genitive forms when the degree of animacy of the possessor is manipulated. For example, animacy may be treated as a binary category [+/−human] or subcategorized further into “human,” “animal,” “company,” “time,” and “place.” The results of these studies show that animate possessors are more likely to be expressed by the *s-*genitive, while inanimate possessors are more likely to be realized with the *of-*genitive. Similarly, experimental studies such as Rosenbach (2001, 2003) show that the higher the referent is in animacy (e.g., human > animal > object), the more likely it is to occur as an *s-*genitive.

Although extensive research has been conducted on the role of animacy in genitive variation in English monolingual adults, the developmental process of how children acquire this linguistic constraint is still under-explored. One study by Skarabela and Serratrice (2009) investigated whether adults and 4 year-old English monolingual preschool children are aware of the animacy constraint, by using a picture-description syntactic priming task. Their results from the baseline task reveal that both the children and the adults used more *s-*genitive than *of-*genitive to express kinship relationships (e.g., the girl’s mother > the mother of the girl). This suggests that 4-year old children are aware of the animacy constraint in the choice of the two genitive forms. Moreover, this finding accords with Bannard and Matthews (2008)’s conclusion that English-speaking children are aware of the two genitive forms around the age of four.

Other research (Anschutz, 1997; Rosenbach and Vezzosi, 2000; Rosenbach, 2001, 2003; Hinrichs and Szmrecsanyi, 2007) have suggested that the givenness or topicality of the possessor influences the choice of genitive forms. Rosenbach (2001, 2003) demonstrated that [+givenness] and [+definite] referents have a higher likelihood of being expressed using the *s-*genitive. Thus, for example English native speakers are more inclined to use the *s-*genitive for a definite possessor that has been previously mentioned (e.g., *the woman’s body*), and the *of-*genitive for a first mentioned possessor with an indefinite article (e.g., *the body of a woman*).

Another relevant factor is the semantic relationship between the possessor and the possessum. Rosenbach (2001) offers a binary categorisation of the possessive relationship in semantic terms: (a) prototypical relationships which consists of kin terms (e.g., *doctor*’*s son*), body parts (e.g., *girl*’*s hand*) and permanent ownership of concrete things (e.g., *father’s car*), and (b) non-prototypical relationships, which cover the remaining possessive cases, including social relations (e.g., *Saint Paul’s teacher*), mental/physical states (e.g., *the girl’s excitement*) and abstract possession (e.g., *the man’s name*) (p. 279). Prototypical relationships have a higher likelihood of being expressed by the *s-*genitive (i.e., [+proto]), while non-prototypical relationships are more likely to be realized by the *of-*genitive (i.e., [−proto]).

**FIGURE 1:**
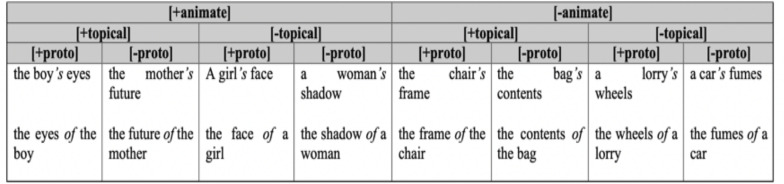
Genitive framework from Rosenbach (2001).

A central concern of the literature has been to tease apart the interplay of these factors to determine which have the greatest and which the least influence on the choice of genitive form (Rosenbach, 2014). The framework established by Rosenbach (2001) tested the relative influence of the factors by combining the three factors (animacy, topicality, and possessive relationship) in a hierarchical structure of cells. The summary of the framework is provided in Figure 1.

Here, animacy is ranked at the top as the primary factor, followed by topicality, and then the type of possessive relationship. Under this framework, the relative frequency of the *s-*genitive is expected to gradually decrease from the far-left condition [+animate][+topical][+proto] to the far right [−animate][−topical][−proto] and vice-versa for the *of-*genitive. Rosenbach (2001) conducted an empirical study on 56 British native speakers of English to test the validity of this hierarchy. She created a forced-choice task between *of-* and *s-*genitive, controlling for the number of examples for each condition and for the other possible factors that might influence genitive choice. The results confirmed her prediction: there was a steady decrease in the proportion of *s-*genitives from the left to right along the cases defined by this framework.

In a questionnaire-like elicitation task, Rosenbach (2003) counted the frequencies of genitive forms from American and British speakers of English. Their main finding revealed that*−*similar to the results of the forced choice task by Rosenbach (2001)–there was a steady decrease of both speakers’ use of *s*-genitives from [+animate][+topical][+proto] to [−animate][−topical][−proto]. It also found that older American-English speakers used more *s*-genitives than older British-English speakers, however, there was no significant difference in the relative frequency of *s*-genitives between the younger American-English and British-English speakers. The finding that the use of *s*-genitive with inanimate nouns is more pronounced in American English than British English is supported by several studies (Rosenbach, 2001; Hinrichs and Szmrecsanyi, 2007; Szmrecsanyi and Hinrichs, 2008), however the majority of these studies have examined press language and so how this variation across varieties of English generalizes to the spoken language still remains uncertain.

Although studies on acquisition of English genitive structure in L2 learners are somewhat limited, a corpus-based study by Gries and Wulff (2013) found that learners’ genitive choice (between *s*-genitive and *of*-genitive) are moderated by their L1. While Chinese speakers used English genitive forms similarly to their English monolingual counterparts, German speakers showed stronger bias toward the use of *s*-genitives. However, Ghilzai (2014) using a speeded judgment task found that German speakers used less *s*-genitives than the monolingual controls, specifically in the [+animate][+topical][+proto], [+animate][+topical][−proto] [+animate][−topical][+proto] conditions as described in the Figure 1 above. Such contradictory results, however, may be an artifact of the methodologies used in these studies−Gries and Wulff (2013) used a corpus and examined linguistic production while Ghilzai (2014) investigated interpretation/comprehension through eliciting judgements.

As mentioned above, it is important to note here that interpretation and processing of English genitives require integration of multiple factors including pragmatic (topicality) and semantic (animacy and prototypicality) information, which are hypothesized to be variable and open to the effects of CLI to different extents (Sorace, 2011, 2016).

### Genitive Structure in Japanese

While English has two genitive constructions, with the choice between them influenced by various factors as discussed in the previous subsection, Japanese has only one construction: the pre-nominal *no* construction. The genitive case marker *no* stands between the possessor and the possessum (e.g., “Hanako *no* penn” “Hanako’s pen”) and thus the construction has a similar linear order to the *s-*genitive in English. There have been argued to be more than fifteen types of semantic relationship that can hold between the two noun phrases in the Japanese genitive construction (Teramura, 1991). Importantly, the Japanese *no*-genitive maps straightforward onto the *s-*genitive in English and both are cliticized morphological exponents right-attached to the possessor noun phrase.

Japanese children start producing the *no-*genitive at an early age (2;2−2;4), and in fact *no* is one of the earliest case particles that they acquire (Clancy, 1985). According to the systematic review of acquisition order of grammatical morphemes in Luk and Shirai (2009), Japanese learners of English acquire the *s-*genitive construction at an earlier stage than other grammatical morphemes such as articles, past-tense morpheme in regular verbs, and third person singular -*s*. This finding has been obtained in studies with Japanese-English children (Hakuta, 1976) as well as adults (Nuibe, 1986; Shirahata, 1988; Izumi and Isahara, 2004). The authors conclude that the linear similarity between the English *s-*genitive and Japanese *no*-genitive allows for positive L1 transfer to occur from Japanese to English.

### Typological Differences Between Japanese and English

Several typological differences exist between Japanese and English. Most importantly, Japanese is an SOV language while English is SVO. Relatedly, English is a head + modifier language in which extensive expansion generally occurs to the right of the non-expandable element, while Japanese is a modifier + head language, where extensive expansion occurs to the left of the non-expandable element. Additionally, English is a mildly synthetic language, while Japanese is analytic in the sense that it has no noun inflection but has a complex system of verb inflection.

## Study 1

Our study first examines bilinguals’ (Japanese-English) and monolinguals’ (English) knowledge of English genitive constructions. In addition, we also examine their knowledge of the word order between the verb and its arguments (subject, object, and indirect object). The choice of the order of the verb and its arguments, specifically in the contexts we use, makes an ideal comparison to the genitive form, as the verb/argument order sentences used in our study lack structural overlap between English (SVO) and Japanese (SOV) and the choice between them is relatively insensitive to interface conditions of an external nature. Thus, the prediction, under all accounts, is that this aspect of word order will not be (easily) affected and thus these conditions serve as a controlled baseline. In Study 1, we first look at preferences for genitive structure (*s-*genitive versus *of-*genitive) and for verb/argument orders, comparing the Japanese-English bilinguals to English monolinguals to see whether there are any differences in their evaluation of these constructions, and if so, in what contexts. The research questions for Study 1 are as follows:

(1) Are there any differences in the evaluation of genitive forms and verb/argument order between bilingual and monolingual children?
(2) If so, does an account of CLI based on linguistic properties suffice to explain observed differences?

First, we do not expect monolinguals and bilinguals to behave differently in their responses for verb/argument order. This is because these aspects of verb/argument order in Japanese (SOV) and English (SVO) do not exhibit structural overlap (Müller and Hulk, 2001) and are largely unaffected by non-syntactic factors (Sorace and Filiaci, 2006). We would expect that the default verb/argument order for both languages was acquired by the onset of testing. However, for the genitive items, as described previously, the children need to know that (a) English has two forms to express possession while Japanese only has one and (b) there are multiple non-syntactic (pragmatic and semantic) factors that influence the choice of the two English genitive forms. The genitive conditions are thus hypothesized to be vulnerable to CLI^1^ creating a context in which non-native like outcomes for bilinguals are expected. Thus, we hypothesize that the bilinguals will behave differently from the monolinguals in their evaluation of genitives.

In order to assess their preference for genitive forms, we used the framework established by Rosenbach (2001) as discussed earlier. To make the experiment manageable for children, we used four out of the eight conditions in the framework. Specifically, we restricted ourselves to the two conditions on the far left and the two conditions on the far right of the genitive framework in Figure 1: [+animate] [+topical] [+proto], [+animate] [+topical] [−proto], [−animate] [−topical] [+proto], [−animate] [−topical] [−proto]. The test conditions and items in our study are discussed in detail in the methodology section. We predict that overextension of *s-*genitives will be manifested in all conditions, not least because if Japanese were to exercise some influence, the *s*-genitive is the only form that overlaps structurally with the corresponding Japanese construction, at least at the surface, given its linear order.

### Methodology

#### Participants

##### Bilingual group

The bilingual group consisted of 36 Japanese-English bilingual children (21 female; 15 male), who acquired English as a second language in a native-English speaking environment outside Japan. The average age of the bilinguals was 9;8 (range 7;6−13;0, *SD* = 1.42). All of the bilingual participants had very minimal exposure to English before leaving Japan. All of the bilingual children’s parents speak Japanese as their native language and the children were exposed to Japanese from birth. Thus, the age of onset of L2 acquisition was the point at which the bilinguals moved to the foreign environment: the average was 5;0 (range 1;0−9;6, *SD* = 2.5). The average length of residence in the foreign country was 4 years (range 2;0−9;9, *SD* = 2.0). Unlike typical Japanese children, the participants learned English through living in a foreign country and attending schools with English as a medium of instruction. Seventeen participants spent their time away from Japan in a country where English is the majority language (United States: *N* = 11, United Kingdom: *N* = 5, Canada: *N* = 1), and the other 19 participants attended international schools in countries where English is not the official language (Netherlands: *N* = 1, France: *N* = 2, Singapore: *N* = 5, Thailand: *N* = 1, France: *N* = 2, Israel: *N* = 1, Malaysia: *N* = 2, Vietnam: *N* = 1, Indonesia: *N* = 1, China: *N* = 2, Poland: *N* = 1), but still received education in English. Although the latter group was exposed to a third language other than Japanese and English, none of the parents reported that their children could actually hold a conversation in the third language. The bilingual participants were recruited from an English maintenance course offered from JOES (Japan Overseas Educational Services). We administered the Bilingual Language Experience Calculator (BiLEC; Unsworth, 2016) to elicit language background information about the bilinguals, which will be further discussed in the context of Study 2 when we examine the change in bilinguals’ syntactic preferences over time.

##### Monolingual group

The monolingual group consisted of 35 children (Mean age = 9;4, range 7;0–13;9, *SD* = 1.6, 15 female). The monolingual children spoke English as their L1 and had very minimal exposure to any L2 (only in language classes at school once a week). The monolingual group was matched to the bilingual group in terms of age and socio-economic status (SES), which was measured by the mother’s final education. All the mothers of the children who participated in this research were educated to Bachelor level or higher. The monolinguals were recruited in Edinburgh, United Kingdom and the majority of them were exposed to British English.

#### Instruments

An untimed, binary forced-choice task was developed by the researchers. The task consisted of 16 genitive items and 16 verb/argument order items. We describe the genitive items first. As mentioned earlier, four out of the eight possible combinations of factors defined in Rosenbach’s (2001) framework were used as conditions in this study: [+animate][+topical][+proto], [+animate][+topical][−proto], [+animate][−topical][+proto], [+animate][−topical][−proto]. We only used four conditions since having four items for each of eight conditions (32 genitive items in total) would have resulted in a long task that would have been too demanding for the children.

**FIGURE 2:**
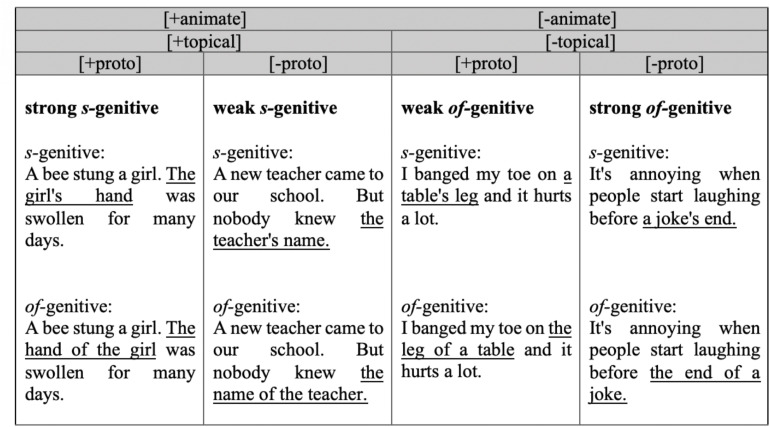
Examples of genitive items.

Examples of each condition are presented in Figure 2 (see Supplementary Data Sheet 1: Table 1 for all items). In the [+animate] [+topical][+proto] example, the possessor is animate (i.e., *girl*) and topical (mentioned in the previous sentence). Further, the relationship between the possessor and the possessum (i.e., *hand*) is prototypical as it expresses the inalienable possession of body parts. In the [+animate][+topical][-proto] example, the possessor is animate and also topical, but the relationship is non-prototypical as the possessum is an abstract object (i.e., a name). The same logic applies for the other two conditions. For convenience, we will label the [+animate][+topical][+proto] as “strong *s-*genitive,” [+animate][+topical][−proto] as “weak *s-*genitive,” [−animate][−topical][+proto] as “weak *of-*genitive,” and [−animate][−topical][−proto] as “strong *of-*genitive” conditions. In the “strong” conditions, all factors favor the same form, whether *s*-genitive or *of-*genitive; in the “weak” conditions the prototypicality factor has the value that influences in the opposite direction to the other two (animacy and topicality).

**TABLE 1:** Summary of BiLEC variables split by language and time; “Abroad” indicates percentage of language exposure when the children lived in the English majority language environment and “Japan” indicates percentage of exposure upon returning to Japan.

	**L2 English**		**L1 Japanese**	
	**Abroad**	**Japan**	**Abroad**	**Japan**
Mean	46.8	4.5	53.2	95.5
SD	12.1	3.2	10.8	8.5
Min	26.5	0	17.5	28.0
Max	82.4	20.5	61.0	92.4

**FIGURE 3:**
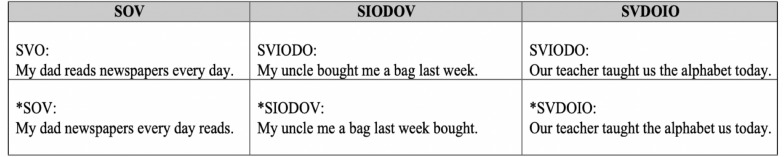
Examples of verb/argument order items.

The verb/argument order items were grouped into three conditions^2^, including paired structures with one grammatical and one ungrammatical order. The ungrammatical sentences were created by manipulating the position of the subject, verb, object and (where present) indirect object (O: object; DO: direct object; IO: indirect object) as illustrated in the examples in Figure 3 (see Supplementary Data Sheet 1: Table 2 for all items). These verb/argument orders (with the exception of SVDOIO) are grammatical in Japanese.

**TABLE 2:** Summary of verbal fluency performance split by language and time; “Round 1” indicates children’s performance at first round of testing and “Round 2” indicates their performance at second round of testing.

	**L2 English**		**L1 Japanese**		**Relative Proficiency**	
	**Round 1**	**Round 2**	**Round 1**	**Round 2**	**Round 1**	**Round 2**
Mean	13.60	15.6	15.40	17.71	1.81	2.38
SD	4.22	3.36	4.99	5.32	4.89	5.39
Min	8	7	8	10	8	15
Max	27	22	34	31	−12	−8

*Relative proficiency is the difference between Japanese and English performance.*

#### Procedure

Two puppets, a male and a female, were presented on a PowerPoint screen. Each puppet read the target sentence using either the *of-* or *s-*genitive structure for the genitive items. For example, the female puppet would say: “*A room’s darkness can make little children scared*”, whereas the male puppet would say: “*The darkness of a room can make little children scared*.” The same procedure was taken for the verb/argument order items. The sentences spoken by the male puppet were recorded by a male native speaker of American English, whereas the female puppet was voiced by a female native speaker of British English. We used speakers of different dialects since some children in the bilingual group were educated through the British system, while others attended schools with an American educational system.

**FIGURE 4:**
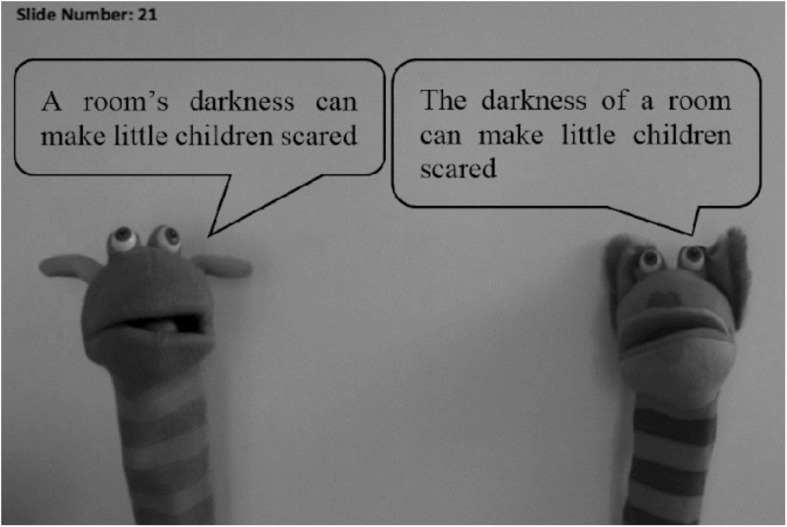
PowerPoint presentation of the forced-choice task.

All participants were seen individually by the researcher in a quiet room, either at home or at school. They were placed in front of a computer screen with the PowerPoint presentation as in Figure 4. They were then asked to listen to the pre-recorded instructions and have one practice trial. During the practice trial, they were asked to choose the puppet that spoke better English. The children were reminded to not base their decisions on phonological factors such as accent or pronunciation. In the practice trials, they were asked to explain their decisions, and if the children’s explanations were related to phonological factors, they were reminded again to focus on what the puppet actually said, and not on how he/she said it. They were also allowed to hear the sentences again if they wished to, but not more than twice. Following the practice trial, 32 trials (16 genitive and 16 verb/argument order) were presented in random order. All of the responses were recorded on paper by the investigator. The position of the puppets (i.e., left or right of the screen), the puppet that started speaking first, and the amount of *of-* and *s-*genitive sentences spoken by each puppet were all counterbalanced.

#### Analysis

In order to examine whether there are differences in the choice of genitives and verb/argument order between monolinguals and bilinguals, we constructed two models using Generalised Linear Mixed Effect Model (GLMER) with logit link. Both models included binary response as a dependent variable and Group (Bilingual, Monolingual) and Condition (genitive model: strong *s*-genitive, weak *s*-genitive, strong *of*-genitive, weak *of*-genitive; verb/argument order model: SOV, S-IO-DO-V, S-V-DO-IO) as predictors. For the genitive model, *s-*genitive responses were coded as 1 and *of-*genitive as 0. For the verb/argument order model, SVO and S-V-DO-IO were coded as 1 and others as 0. We included Subject and Item as random intercept (adding by-Subject and by-Item random slope for Condition did not improve the overall fit of the model). For the genitive model, the reference level was set to “bilinguals” for the Group and “strong *of*-genitive” for the Condition variables. For the verb/argument order model, the reference level was set to “bilinguals” for the Group and “SOV” for the Condition variables.

#### Results

##### Genitive form

**FIGURE 5:**
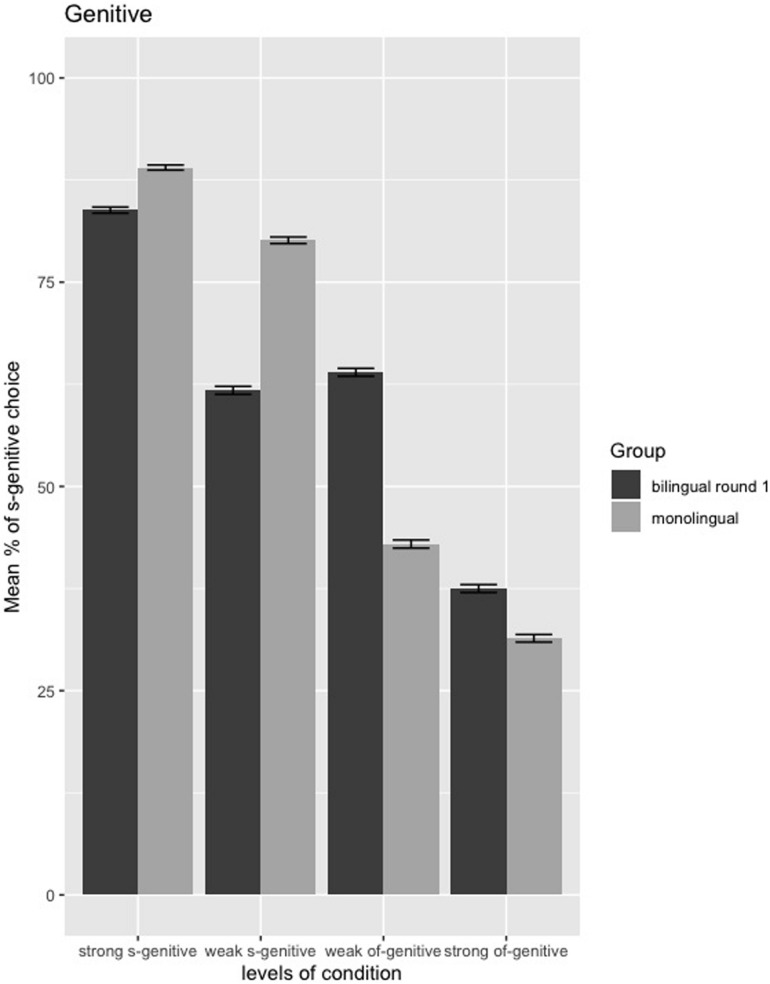
Mean percentages of *s*-genitive choice in the genitive forced-choice task between monolinguals and bilinguals from first round of testing; error bars = standard error.

The estimated coefficients of the genitive model are presented in the Supplementary Data Sheet 2: Table 1. As shown clearly in the comparison of mean percentages of *s-*genitive choice between monolinguals and bilinguals from the first round of data collection (Figure 5), significant differences in the evaluation of genitive forms were found for two conditions: the weak *of*-genitive condition and the weak *s*-genitive condition. Pairwise comparison (Tukey’s test) demonstrates that the bilinguals used more *s-*genitives in the weak *of-*genitive condition than the monolinguals (*E* = 0.88, *SE* = 0.24, *z* = 3.56, adjusted *p* = 0.008). In contrast, the monolinguals’ preference for *s-*genitive was higher than the bilinguals in the weak *s-*genitive condition (*E* = −0.99, *SE* = 0.27, *z* = −3.58, adjusted *p* = 0.008).

##### Verb/argument order

**FIGURE 6:**
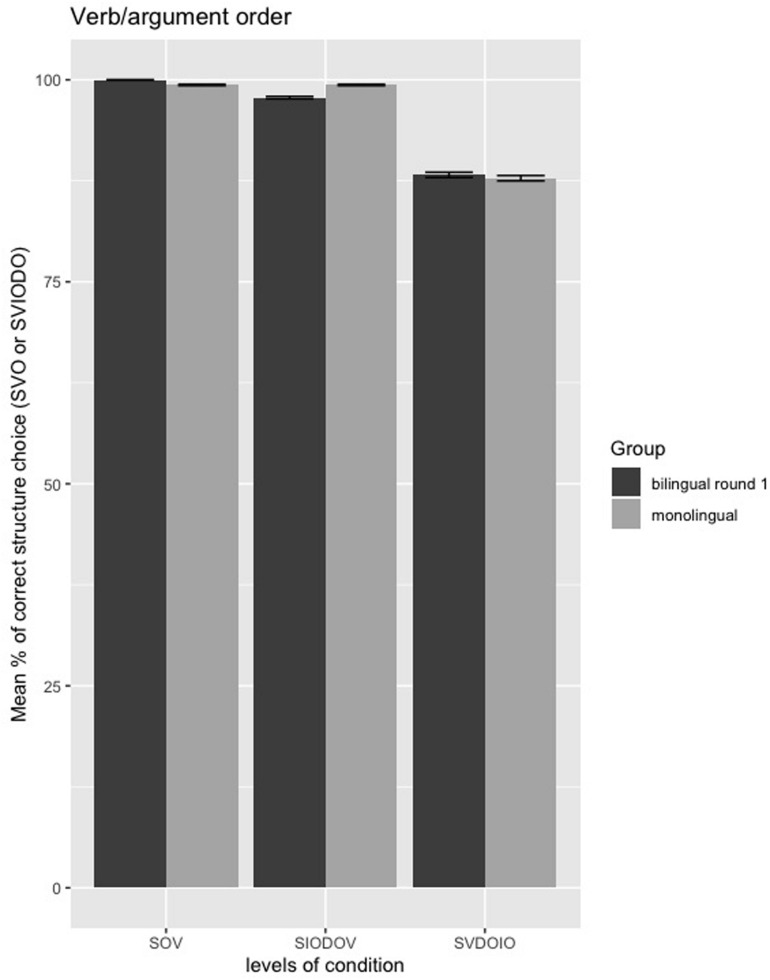
Mean percentages of correct/preferred structure choice (SVO or SVIODO) in the verb/argument order forced-choice task between monolinguals and bilinguals from first round of testing; error bars = standard error.

The estimated coefficients of the verb/argument order model are presented in the Supplementary Data Sheet 2: Table 2. There are no significant differences between monolinguals and bilinguals in their evaluation of verb/argument order, given the lack of significant interactions between Group and Condition (*p*’*s* > 0.79). Moreover, as shown in Figure 6, the performance on all verb/argument order conditions was at near-ceiling (SVDOIO) or at ceiling (SOV, SIODOV) for both monolinguals and bilinguals.

#### Discussion

In Study 1, we predicted that bilinguals would behave differently from monolinguals in their evaluation of the two types of genitives, but not in their evaluation of the different verb/argument orders. More specifically, it was expected that bilinguals would over-extend *s-*genitives compared to their monolingual counterparts. As predicted, bilinguals and monolinguals did indeed behave similarly on their evaluation of verb/argument order; however, our results do not bear out the predictions pertaining to the genitive entirely. Recall that the bilinguals not only preferred to use *more s-*genitive in the weak *of-*genitive condition, in line with our predictions, but they also chose *less s-*genitive in the weak *s-*genitive condition when compared to the monolinguals. That is, bilinguals behaved differently from monolinguals in the two “weak” conditions*−*those for which monolinguals weakly favor either the *s-*genitive or the *of-*genitive, respectively−but did not differ in the “strong” conditions.

The fact that the monolinguals were predominantly exposed to British English in the United Kingdom, while the bilingual group were exposed to different varieties of English dialects may have had some effects on the observed differences in genitive preference between monolinguals and bilinguals. Given that the use of *s*-genitive with inanimate nouns has been found to be more common in American English than British English (Rosenbach, 2001, 2002, 2003; Hinrichs and Szmrecsanyi, 2007; Szmrecsanyi and Hinrichs, 2008), one could expect monolinguals (who were mainly exposed to British English) to choose less *s*-genitives with inanimate possessor (i.e., weak *of*-genitive and strong *of*-genitive conditions) than the bilinguals, in which at least one-third of them were predominantly exposed to American−English. Our results show that indeed, bilinguals used *more s*-genitive in the weak *of*-genitive condition than the monolinguals but behaved similarly in the strong *of*-genitive condition. Moreover, dialectal differences cannot account for the differences in the weak *of*-genitive condition, as the bilinguals preferred *less s-*genitive in the weak *s-*genitive condition. The limitation of this study is that the monolingual group was homogenous in terms of the English dialect they were exposed to, which makes it difficult to tease apart the influence of dialectal differences vs. CLI from the native language. Future studies should either keep the English dialect consistent between two groups or include speakers from various English dialects for both groups when comparing the genitive form use/interpretation between monolinguals and bilinguals.

These results suggest that bilinguals were able to choose the “preferred” genitive form in the “strong” conditions, regardless of whether it is a context that induces a strong preference for the *s-*genitive or for the *of-*genitive. That is, in the conditions that have consistent [+factors] or [−factors] (i.e., strong *of-*genitive: [−animate][−topical][−proto] or strong *s-*genitive: [+animate][+topical][+proto]), the bilinguals are not different from the monolinguals in their choice of genitives. That is, in these contexts*−*the polar ends of Rosenbach’s continuum*−*the relevant feature configurations provide the strongest cluster sets biasing one or the other form. Recall that in all conditions both the *s*-genitive and *of*-genitive are grammatical. What is at stake is their relative likelihood of being chosen. Since bilinguals perform, in general, closer to monolinguals when optionality is reduced, this could account for the data we obtain. In other words, if there is a continuum of optionality (stronger versus weaker conditions in Rosenbach’s terms), we should note less bilingual divergence where things appear more categorical as is the case when the relevant set of feature values is at one or the other end of the continuum. However, when one factor (prototypicality) is in conflict with the other factors (topicality and animacy) as in the weak conditions (weak *of-*genitive: [−animate][−topical][+proto] or weak *s-*genitive: [+animate][+topical][−proto]), the bilinguals appear to have more difficulties in selecting the preferred structure in line with the monolinguals as the evaluation of the feature set is more complex and requires more processing resources. Taken together, the findings do not indicate an overextension of *s-*genitives *per se*, but rather demonstrate that the bilinguals behave differently from monolinguals in conditions that require processing of conflicting semantic and pragmatic factors. Consequently, this suggests that the differences in the choices of highly proficient bilinguals (recall they were tested soon after on average 4 years of immersion in a native English environment) and monolinguals cannot solely be attributed to CLI due to the internal structure of the two languages, but, in this case, also depends on the relative complexity of semantic and pragmatic integration, consistently with the Interface Hypothesis (Sorace, 2016).

## Study 2

Study 2 examines the same bilingual children a second time, comparing Round 2, one year after the first testing, to their own performance from Round 1 (on average within the first weeks of returning to Japan). The results are compared to determine whether there is any change in the choice of genitive forms and verb/argument order, using each bilingual individual as their own baseline. The research questions for Study 2 are as follows:

(3) Are there any changes in the bilingual returnee children’s evaluation of genitive forms and verb/argument orders over time?
(4) If so, can the observed change be explained by CLI from the dominant to the non-dominant language?

If CLI in the genitive structure is (partially) due to language dominance, then the prediction is that there should be unidirectional CLI from L1 Japanese to L2 English in bilingual returnee children that will increase over time after return to the Japanese environment. This would be due to their L1 Japanese becoming increasingly more dominant after their return to the L1 environment. Since the Japanese *no*-genitive resembles the linear order of *s-*genitive in English and both are cliticized morphological exponents right-attached to the possessor noun phrase, we expect the preference for *s-*genitives to increase across the four conditions. In terms of verb/argument order, there are two possible predictions. If language dominance alone is “enough” for CLI to occur irrespective of the nature of the underlying structural representation, then we expect children to also accept more erroneous verb/argument order (e.g., SOV) in English over time. However, if some degree of (ambiguous) structural overlap of linguistic properties across the two languages and language dominance are necessary for CLI to take place (Müller and Hulk, 2001) or if basic word order is particularly resilient, then it would be expected that change in language dominance would only affect genitive forms, but not verb/argument order.

### Methodology

#### Participants

##### Bilinguals

Of the original group of 36, two participants’ data were not recorded due to technical issues, leaving 34 in the second round of testing. The average time that elapsed between Round 1 testing and Round 2 was 12 months (range 10−13 months, *SD* = 0.64). Recall that in the first test session, the average age of the bilinguals was 9;8 (range 7;6−13;0 years, *SD* = 1.42). The average age at time of testing in Round 2 is 10;8 (range 8;6−14;0 years, *SD* = 1.42), reflecting a true year between testing sessions.

As mentioned in the Methodology section in Study 1, the Bilingual Language Experience Calculator (BiLEC; Unsworth, 2016) was administered to the parents twice in order to elicit information about quantitative language exposure of the bilingual children in each language and history of language use. The first administering was done at the time of the first testing session and focused on exposure and use of Japanese and English during the stay abroad. The second focused on the distribution of Japanese and English since the time of return. Bringing the two together, it is possible to measure the change at the individual and group level of exposure and engagement with both languages upon return to Japan. The relevant information is summarized in Table 1 above.

As shown in Table 1, the children’s average English exposure decreased from 46.8% while abroad to 4.5% upon return to Japan, a drop of 42.3%. Their Japanese exposure at home and at school increased in an inverse proportion (from 53.2% to 95.5%). It is important to point out here, the bilingual children were not “English-dominant” even when living abroad, since they received around half of their exposure in English and the other half in Japanese. Rather they were receiving balanced L1 and L2 exposure. However, when returning to Japan, we can see that the exposure they received was clearly Japanese-dominant, with having 95.5% of their exposure in Japanese.

#### Instruments and Procedure

We used the same forced-choice task in Study 1 to examine the changes over time in the bilingual children’s preferences for genitive and verb/argument order. The only difference in the task was that lexical changes were made for each item in order to reduce learning effects in the second round of testing. For example, “*The darkness of a room/a room’s darkness can make little children scared*” was changed to “*The darkness of a room/a room’s darkness can make people anxious*.” The target phrase (e.g., *the darkness of a room/the room’s darkness*) remained the same in the first and the second test. In the second test, a female native speaker of Canadian English voiced the female puppet and a male native speaker of British English the male puppet, since some children were educated through the British system and others American. The procedure was exactly the same as the first round of testing.

In order to measure the bilingual children’s relative proficiency, we administered a verbal fluency task at both first and second round of testing for the bilingual children. The participants were asked to name either (1) animals or (2) fruits and vegetables in English or Japanese. Half of the bilinguals named animals in English and fruits and vegetables in Japanese, and vice-versa for the other half of bilingual participants. For all participants, a timer was set to 1 min by the researcher and the participants were all given approximately ten seconds after they listened to the instruction to start the task. Their responses were recorded on a voice-recorder and later transcribed by two research assistants. The total number of unique words was calculated for each participant, and the difference between Japanese and English scores for each participant was used as a measure for their relative proficiency. The higher the values are, the more proficient they are in Japanese and vice versa for English.

#### Analysis

We constructed two GLMER models: one with genitive responses and the other with verb/argument order responses. In the genitive model, we included binary response as a dependent variable and Time (Bilingual Round 1, Bilingual Round 2), Condition (genitive model: strong *s*-genitive, weak *s*-genitive, strong *of*-genitive, weak *of*-genitive), Age of L2 onset (AoO)^3^, L2 exposure difference (i.e., the difference in L2 exposure when they lived in an L2 majority language environment vs. back in the L1 environment), and Relative proficiency (at first round of testing) as predictors. Each language background variables (i.e., AoO, L2 exposure difference, Relative proficiency) were included as a three-way interaction between Time and Condition, in order to examine whether the change in genitive preference for each condition is affected by bilinguals’ language experience. We did not include background variables as predictors for the verb/argument model, since we did not expect these variables to influence the change of preference in verb/argument order. In addition, because the bilingual children performed at ceiling for first and second round of testing on the verb/argument items, including a three-way interaction among Condition, Round, and the three background variables resulted in model conversion errors.

For the genitive model, *s-*genitive responses were coded as 1 and *of-*genitive as 0. For the verb/argument order model, SVO and S-V-IO-DO were coded as 1 and others as 0. For the genitive model, the reference level was set to “Bilingual Round 1” for the Time and “strong *of*-genitive” for the Condition variables. For the verb/argument order model, the reference level was set to “Bilingual Round 1” for the Time and “SOV” for the Condition variables.

#### Results

##### Relative proficiency

The results of their verbal fluency performance at first and second round of testing is presented in Table 2.

Children performed better in Japanese than English for both first and second round of testing, and they increased their scores from first to second round of testing for both English and Japanese. They also appear to have relatively balanced L1 and L2 proficiency, although their dominance in Japanese proficiency increases from first to second round of testing. A categorical classification of relative proficiency is determined through calculating whether there is a difference greater than 1 standard deviation between Japanese and English scores (Unsworth, 2003). Following this categorization, 4 children were English-dominant, 13 children were Japanese-dominant, and 19 children were balanced bilinguals in the first round of testing. In the second round of testing, only 1 child was English-dominant, 10 children were Japanese-dominant, and 25 children were categorized as balanced bilinguals.

##### Genitive form

**FIGURE 7:**
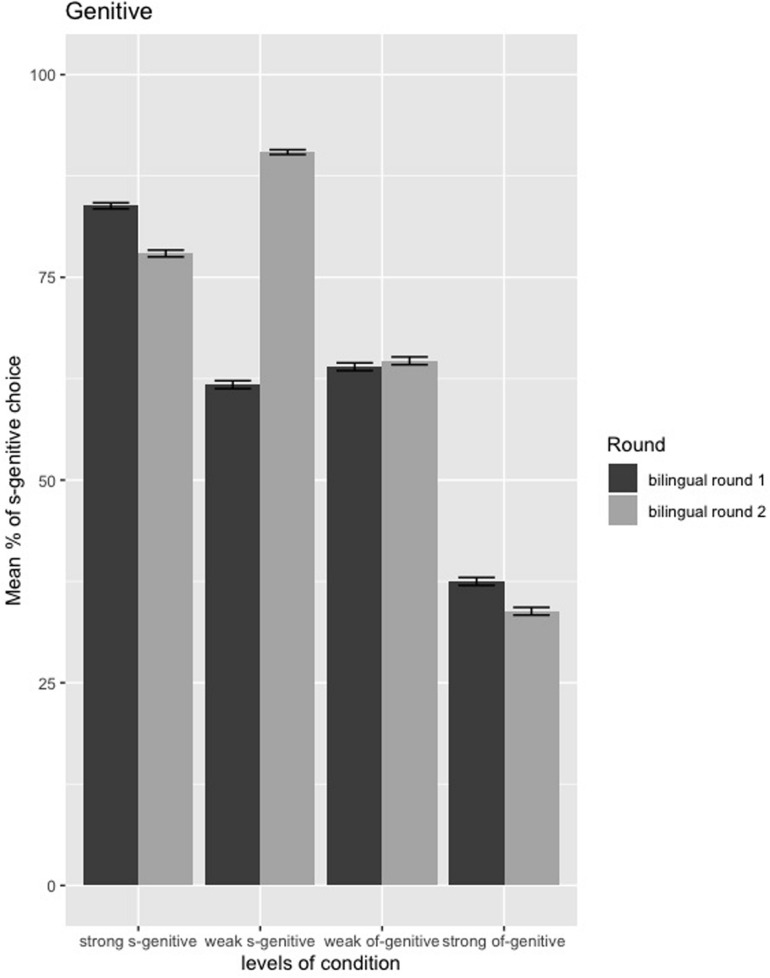
Mean percentages of *s*-genitive choice in the genitive forced-choice task between first round and second round of testing in bilinguals; error bars = standard error.

The model summary (see Supplementary Data Sheet 2: Table 3) shows that there is an interaction between Group and Round (*E* = 2.50, *SE* = 0.54, *z* = 4.62, *p* ≤ 0.001), and the pairwise comparison (Tukey’s test) reveals that there was a difference in the genitive evaluation from first to second round of testing in the weak *s*-genitive condition only (*E* = −1.85, *SE* = 0.34, *z* = −5.34, adjusted *p* ≤ 0.001). The bilinguals showed a greater use of the *s-*genitive in the weak *s-*genitive condition after a year spent back in the Japanese environment, as also illustrated in Figure 7.

There were no significant two-way interactions between Round and Background variables (AoO, L2 exposure, and Relative proficiency) (*p* > 0.40) nor a significant three-way interactions among Round, Condition, and Background variables (*p* > 0.08).

**FIGURE 8:**
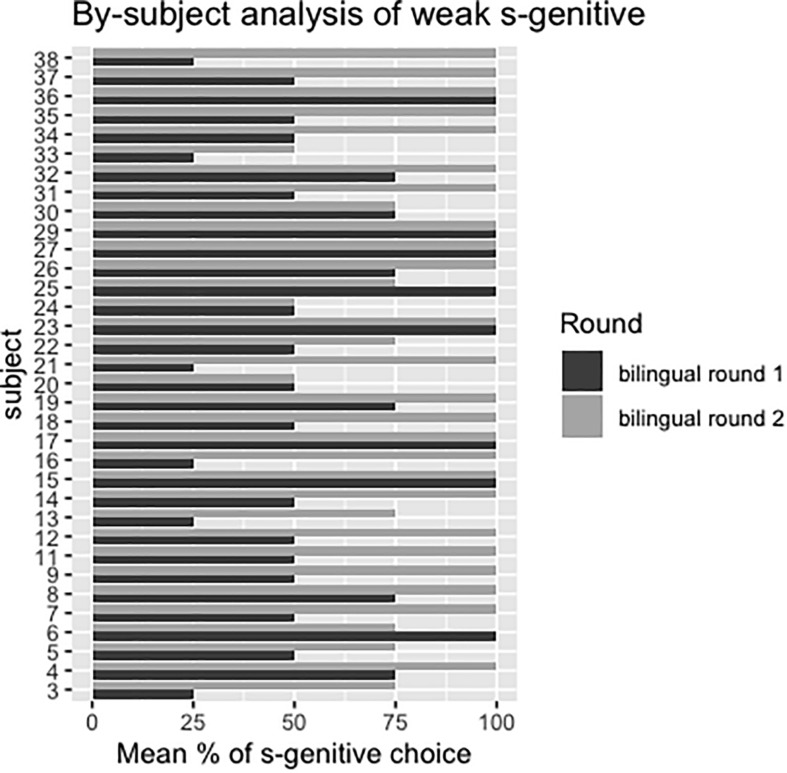
Mean percentages of *s*-genitive choice in the weak s-genitive condition from first to second round of testing for each subject.

**FIGURE 9:**
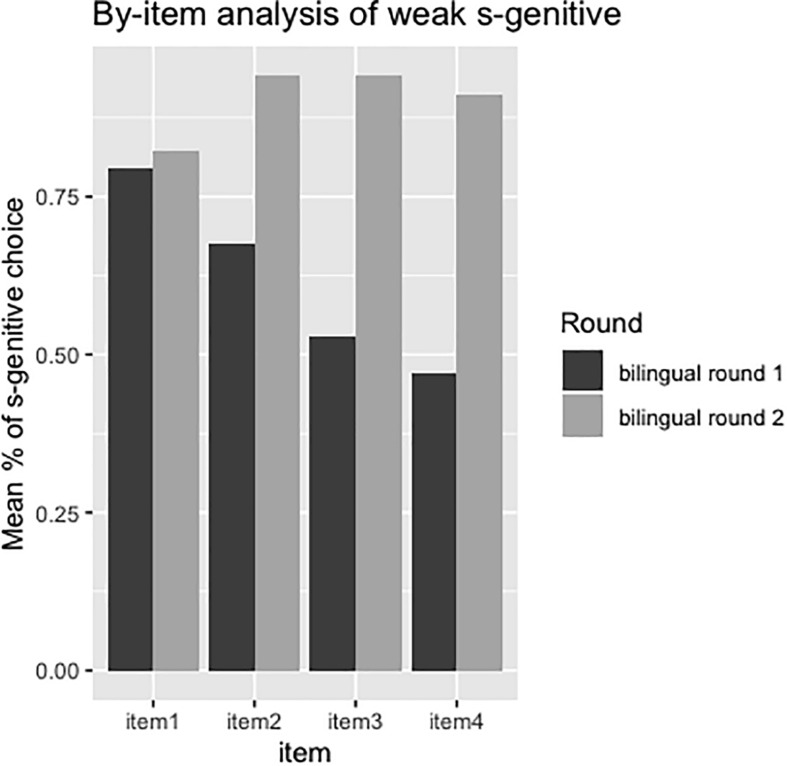
Mean percentages of *s*-genitive choice in the weak s-genitive condition from first to second round of testing for each item.

Since the weak *s*-genitive condition was the only condition in which children’s preference changed significantly over time, we conducted a by-subject (Figure 8) and by-item (Figure 9) analysis on this dataset. Figure 8 shows that only two participants (subject 4 and subject 22) had decreased their proportion of *s*-genitive choice over time. Moreover, while eight participants had chosen *s*-genitive 100% of the time in the weak *s*-condition at the first round of testing, 24 participants had chosen *s*-genitive 100% of the time in the second round of testing. Only three participants (subject 20, subject 24, subject 33) in the second round of testing were at chance-level in choosing *s*-genitive, while others were above chance (either 75% or 100%).

In the item-based analysis in Figure 9, we can see that some items elicited more *s*-genitives than others in the first round of testing, while in the second round of testing, four out of three items (item 2: The teacher’s joke/the joke of the teacher was very mean; item 3: The woman’s voice/the voice of the woman was very loud; item 4: A life guard saved the man’s life/the life of the man) elicited more than 90% of *s*-genitive choices. Item 1 (But nobody knew the teacher’s name/the name of the teacher) was the only one that elicited less than 90% (around 82%) of *s*-genitive choice.

##### Verb/argument order

**FIGURE 10:**
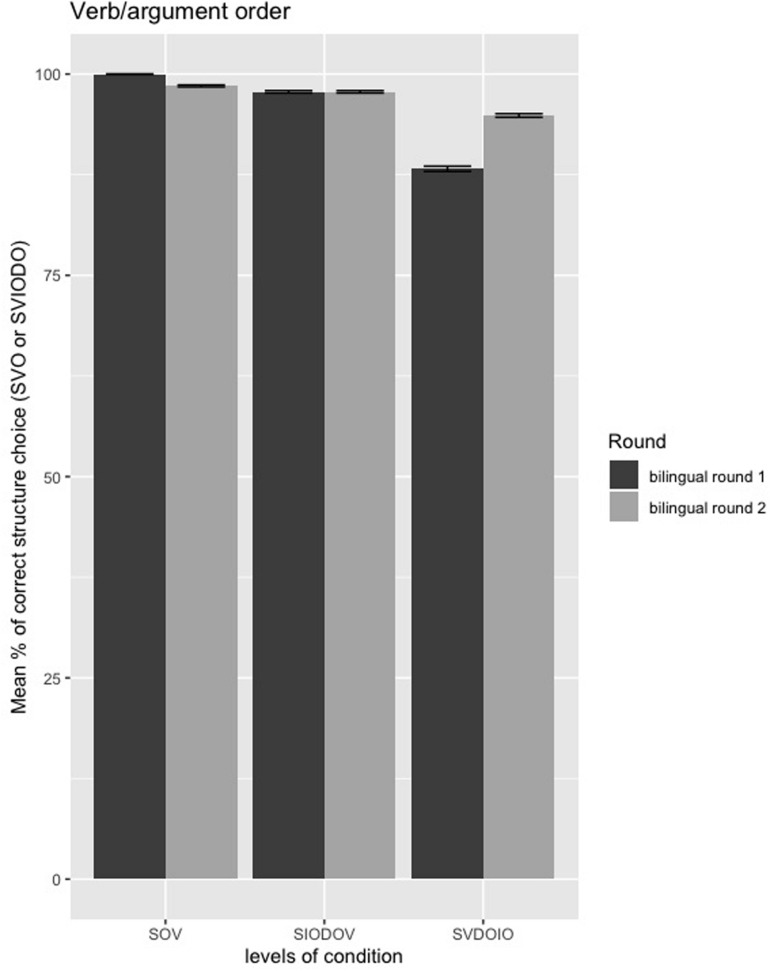
Mean percentages of correct/preferred structure choice (SVO or SVIODO) in the verb/argument order forced-choice task between first and second round of testing in bilinguals; error bars = standard error.

The estimated coefficients of the verb/argument order model are presented in Supplementary Data Sheet 2: Table 4. The model output shows that there are no significant differences within bilinguals over time in their evaluation of verb/argument order, given the lack of significant interactions between Group and Round (*p*’s > 0.08). As shown in Figure 10, the performance on all verb/argument order conditions remained at ceiling over time (and behaved more closely to the monolinguals in the second round of testing). Despite a significant decrease in exposure over time, there appears to be no negative (or positive) changes in the returnees’ evaluation of verb/argument order over time.

#### Discussion

In Study 2, we examined the extent to which the bilinguals’ choice of forms changes over time after the move from an L2-dominant to an L1-dominant environment. If CLI effects are driven entirely by L1 dominance, this would predict an overall increase in the acceptance of non-native-like word orders (e.g., SOV) over time, and similarly an increase of *s-*genitive choice across the genitive conditions. Our results, however, do not support this. First, there was no change in the evaluation of verb/argument order over time. Second, our data show that while the preference for *s-*genitive in the weak *s-*genitive condition did increase from first to second round of testing, it was only in this one condition that we observed an increase in *s-*genitive*−*there was no general increase of *s-*genitives across the board. In sum, these findings suggest that language dominance alone is not sufficient to explain our results: neither the lack of change in evaluation of verb/argument order nor the restricted change in genitive choice. What then can explain the pattern we observed? Does it instead support the other hypothesis we entertained based on a convergence of language dominance and the grammatical status of the linguistic properties tested? Recall, the prediction was that overlap and interface conditions would determine which properties would undergo increased CLI due to significant reduction in L2 input. Under this approach, verb/argument order was expected to remain unaffected, as was borne out in the data. However, this approach also would have predicted a more generalized extension of *s*-genitive, which we did not observe. Thus, this second hypothesis was only partially confirmed, a point to which we return in greater detail below.

## General Discussion

As stated earlier, the aim of this study was two-fold. First, we compared the genitive and verb/argument order choice between bilinguals and monolinguals in order to investigate whether linguistic properties play a role in the presence of CLI. Second, we tracked the genitive and verb/argument order choice of bilingual returnee children over time to test whether change in language exposure has any effect on the evaluation of these two structures.

The findings of Study 1 with respect to genitive choice revealed that bilinguals and monolinguals behaved differently on the “weak conditions” only*−*where prototypicality is in conflict with the other two factors (i.e., animacy and topicality). This finding is not in line with our initial predictions. Recall that we expected there to be an effect for overextending the *s-*genitive, which we predicted would manifest across all conditions. Our results here are instead more in line with the account proposed by Sorace (2011, 2016): namely, that the coordination of multiple factors involved in the process of choosing one structure over another is a more demanding task for bilinguals. In the current study, integrating three factors that govern the choice of genitives*−*animacy, topicality, and prototypicality*−*may be particularly taxing for bilingual children.

In the “strong” conditions, all three semantic and pragmatic factors are aligned. For example, in the strong *s-*genitive condition, *prototypicality*, *topicality*, and *animacy* are all positive valued, resulting in the strongest bias for the *s-*genitive form. When these factors are aligned, bilinguals seemingly have enough resources to process this information in a qualitatively similar way to monolinguals. As a result, monolinguals and bilinguals do not differ in choosing amongst the *s*-genitive and *of*-genitive options. In other words, the absence of conflicting information makes processing easier. However, when one factor is in conflict with the other two, the bilinguals are apparently less efficient at processing conflicting factors relative to monolinguals. The difference in the resolution of conflicting factors, then, gives rise to increased variation and affects the determinacy with which a form is selected.

**FIGURE 11:**
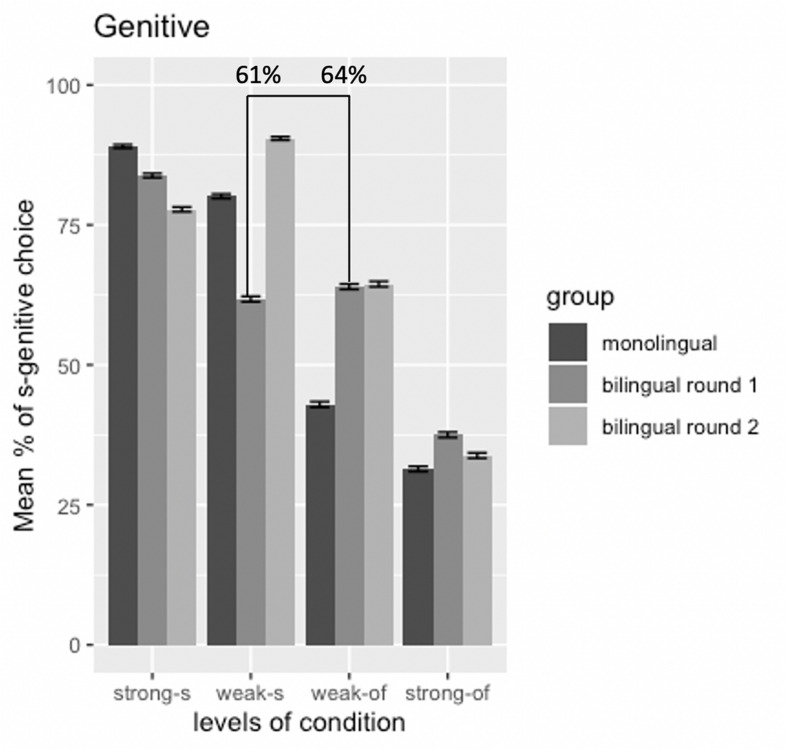
Mean percentages of *s*-genitive choice in the genitive forced-choice task between monolinguals and bilinguals as well as bilinguals from first to second round of testing (highlighting the distributional patterns of the bilinguals in the two weak conditions); error bars = standard error.

As we saw in Figures 5, 9 above, which we combine below as Figure 11 for ease of reference, the above discussion is supported by the distributional patterns of the bilinguals in the two weak conditions.

Two patterns can be noted: (a) in the weak *s-*genitive condition the proportion of *s-*genitive choice shows lower determinacy, gravitating close to chance than the monolingual pattern; (b) in the weak *of-*genitive condition it shows greater determinacy, moving away from chance, but in the direction of a preference opposite to that of the monolinguals. However, crucially in both cases, the pattern of the bilinguals favors the *s*-genitive, the only genitive form in English that shares structural overlap with Japanese. In fact, in both “weak” conditions bilinguals choose *s-*genitives roughly 60% of the time, as indicated in Figure 11 below. Thus, it is not the case that the bilinguals are randomly choosing. In fact, it seems that the bilingual children are treating both “weak” conditions comparably, even though for native speakers the two “weak” conditions display essentially opposite patterns. While the bilinguals show a reduction in *s-*genitive preference in the weak *s*-genitive condition (61% for bilinguals as opposed to 80% for the monolinguals) and an increased preference for *s-*genitive in the weak *of*-genitive condition (64% for bilinguals compared to 43% for monolinguals), both are the consequence of the same underlying issue. Recall that potential influence from Japanese could only favor *s*-genitive selection. For this reason, we see that in both “weak” conditions *s*-genitive is chosen significantly above chance. However, the fact that both conditions converge toward using the *s*-genitive to the same degree suggests that conflicting cues, irrespective of how they manifest in the target grammar, are equally difficult for bilinguals. Nuance between the conditions does not obtain precisely because each has conflicting factors within the set that determines genitive preference choice. Overall, then, increased complexity of conflicting cues creates a context in which bilinguals do not perform like monolinguals, despite clearly having a grammar with both genitive forms. In such a case, CLI effects might be attenuated in favor of a real time choice preferring *s*-genitive to the same degree, irrespective of the context.

According to the Interface Hypothesis, it is not surprising that processing conflicting factors may be more difficult for bilingual children, given that they also need to simultaneously inhibit the non-target language. In other words, allocating general cognitive resources to resolve such conflicts becomes a more demanding task, since only bilinguals have to manage the target language while suppressing the language that is not in use (Green, 1998; Meuter and Allport, 1999; Linck et al., 2009). Further, suppressing the language not in use may be particularly costly when that language is dominant, as is the case here for Japanese. This finding adds a new perspective to the hypothesis that integrating information from multiple domains increases the cognitive load (Sorace, 2011, 2016). Our results show that in addition to this, resolving conflicts between semantic and pragmatic constraints may also be cognitively demanding and thus particularly difficult to accomplish for bilingual children. We should however note that it is entirely possible that “semantic” and “pragmatic” conditions are different in terms of cognitive load, not so much because the former is “internal” and the latter is “external” *per se*, but because of the inherent higher variability of pragmatic conditions, which change in the course of contextual interaction and have to be continuously re-assessed. Our research, however, did not aim to disentangle these different conditions or measure the cognitive load they entail, and this is work that needs to be done in future research.

It may also be worthwhile in future research to investigate other cases where constraints on linguistic structures are in conflict, to examine whether there are other, similar cases of conflicting constraints where we would predict the same type of processing difficulties and behavioral corollaries in performance for bilinguals. Another obvious follow-up would be to examine genitive preference in French-English bilinguals, given that French, like Japanese, has only one genitive construction, but is the “mirror image” of Japanese in that French has only a post-nominal genitive. If processing cost, rather than CLI, explains the divergence between bilinguals and monolinguals, then French-English bilinguals should also behave differently from English monolinguals in their evaluation of genitive conditions that exhibit conflicting factors.

Turning to the verb/argument order conditions, here we found no significant differences between monolinguals and bilinguals. As discussed previously, the items from the verb/argument order conditions were formed by varying the word order of a subject, verb, and an object. This type of verb/argument order involves consideration of syntactic properties insensitive to external conditions. Processing the word order of S, V, O and IO does not require the same amount of cognitive load as genitive forms choice, which require integrating multiple semantic and pragmatic factors. Since the processing task here is relatively easy, bilinguals may have been as effective as monolinguals in parsing syntactic information.

We now turn to the question of whether the results of Study 2 are compatible with what we argued earlier on the basis of Study 1 data, or whether they call for some reconsideration. We note that the data from Study 2 are not compatible with the original hypothesis that the use of *s*-genitive increases across all four conditions. The results from Study 2 are compatible, however, with the general claims made on the basis of Study 1 data, although some qualifications are in order. Recall that in Study 2, there is a longitudinal shift in the bilinguals in one condition only, the weak *s*-genitive. That is, for all other genitive conditions, and all word order conditions, the bilinguals perform in the same way immediately on return to Japan and one year later.

We will deal with the least complicated data first, this being the word order conditions. Recall that the bilinguals in Study 1 were no different from the monolingual controls in any of the word order conditions, which we interpreted as their having acquired the target grammar in English for these core syntactic properties. The fact that there is no change in state over one year of re-immersion in their L1 Japanese in Study 2 provides rather strong evidence that core syntactic properties, once acquired, are resilient to changes in input exposure and relative language dominance in the environment.

Turning to the genitive conditions, three things are of particular significance: (a) the sharp change with the weak *s*-genitive condition, (b) the lack of change for the other weak condition (weak *of*-genitive condition) and (c) the lack of change for both “strong” conditions. The change from 61% to 90% choice of *s*-genitive in the weak *s*-genitive condition indicates clear CLI, not least because the bilinguals are now showing evidence of moving toward making this a categorical choice, going well beyond the monolingual rate of 80% in Study 1. What we see, then, is that the bilinguals’ difficulty in dealing with conflicting semantic and pragmatic cues is replaced by an even larger CLI effect. In other words, it seems that for the bilinguals there is no longer a competition between the *of* and the *s-*genitive in this condition: CLI from Japanese, in this environment of dramatically decreased exposure to the L2 and increased exposure to L1 Japanese, has led to a shift toward categorical choice of the *s-*genitive. However, if this were the whole story, why should the same effect not be evidenced in the other conditions? In other words, while this might be a reasonable explanation for (a) above, it is not enough to explain (b) and (c). Further considerations are therefore in order.

We must keep in mind that, while young in age, the bilinguals tested here were very highly proficient in their L2. After all, it was the majority language of their environment for an average of 4 years of their young lives. With this in mind, we submit that (c)*−*the lack of change in the “strong” conditions*−* is explained because there is effectively no room for Japanese CLI to be manifested in the strong s-genitive condition, since already at the point of return to Japan, in Study 1, the children already highly favored the *s-*genitive. For the strong *of*-genitive condition, given how proficient they are in English, we would not expect that one year would be sufficient to see a change for the extreme polar end of Rosenbach’s scale. As for (b) *−*the lack of change in the weak *of*-genitive condition*−*we should note again where this condition sits on Rosenbach’s scale (Figure 1). Of the three weak *of*-genitive conditions, it is most likely to correlate with *of*-genitive choice. Therefore, we argue that again one year is not sufficient for CLI to have worked down the scale enough to see the same effect that happened for the weak *s*-genitive condition. In other words, we expect that it would take longer for Japanese CLI to overtake the bilinguals’ knowledge of English for the weak *of-*genitive condition because it is further down in Rosenbach’s scale in relation to what is most similar in form to Japanese; *s*-genitive being much closer to the Japanese *no*-genitive (linearly and in terms of its morphological status).

Recall that we presented the choice between *s-*genitive and *of*-genitive in native English in relation to Rosenbach’s scale. There are a total of eight permutations of the three semantic and pragmatic factors (see Figure 1 above), however, we only used four conditions to make the experiments manageable for testing with children. We kept the polar ends of the scale, four conditions (two of each) in which either *s*-genitive or *of-*genitive is predicted to be most likely chosen. Effectively, we eliminated the least clear conditions, ones where the natives would be increasingly more likely to inch toward chance levels in choosing between the competing structures. Had we been able to test all the conditions, we would have been in a position to really address the hypothesis that CLI influence is a gradual process, one that follows in accord with formal descriptions such as Rosenbach’s scale. Given the language pairing and context we are working with, evidence from bilingual populations such as Japanese-English returnees could be used as a unique testing ground for determining its ecological validity. If this account is on the right track, in a situation of massively decreased exposure to English in favor of Japanese, we would expect a change in the direction of increased choice of the *s*-genitive in Japanese-English bilinguals that would be manifested first near the left end of Rosenbach’s scale and then progressively affect the other environments going from left to right; with the weak *of*-genitive condition that we tested only susceptible at a very late stage. This prediction deserves to be tested empirically in further work: this can be done by re-introducing the additional conditions on Rosenbach’s scale that had to be excluded in this study. In summary, then, we submit that the data as a whole from Study 2 suggest that re-immersion increased CLI effects, and that this happened in a gradual, yet principled way (i.e., following from a motivated linguistic hierarchy).

As pointed out by a reviewer, in order see whether there are viable alternatives to the analysis we offered above based on conflicting semantic and pragmatic features, one could investigate the relative frequencies of the forms. That is, it could be the case that what we observed in our monolingual vs. bilingual comparison is not an effect of CLI or difficulties in conflict processing, but rather a result of delayed acquisition of genitive forms due to reduced input frequency. What we would expect from this line of reasoning is that Japanese-English bilingual children are, in fact, still in the process of acquiring the distributional patterns of English genitive forms and are following a similar (but delayed) acquisition pattern to that of typical monolinguals, precisely because the bilinguals differ from them in many aspects of language experience, including quality and quantity of input, age of onset, and length of exposure. The significant role of input frequency in determining similar acquisition patterns has, for example, been attested in the case for Aspect as seen, for example, in Romance languages. Each of the four categories of inherent semantics of verbs or so-called *Aktionsart* (achievement, accomplishment, activity, state) can be characterized in terms of three semantic features: telic, punctual, and dynamic. Past and progressive inflections are initially restricted to certain *Aktionsart* predicates (perfective past on achievement verbs and imperfective past on stative verbs). Over time, monolingual children extend these prototypical forms to other verb types across the scale until eventually both forms can be used with all four verb types in a progressively predictable way based on viewpoint aspect (Andersen, 1991; Andersen and Shirai, 1994). And so, while initially imperfective past is used exclusively with stative verbs (-dynamic, -telic, -punctual) and perfective past with achievement verbs (+dynamic, +telic, +punctual), their prototypes, both perfective and imperfective morphology begin to be used with other verb types with non-aligning semantic features, (i.e., accomplishment and activity verbs). A similar line of reasoning could be applied to the acquisition of genitive forms in English. That is, *s*-genitive and *of*-genitive might be shown to have discernible prototypes. Their use in other contexts might be predicted to align with the progressively weaker semantic feature bundles that give rise to a preference for one or the other form, before eventually, each form can be used in all eight categories described in the Rosenbach’s hierarchy.

Testing this alternative hypothesis would clearly require investigating monolingual English and Japanese-English bilingual child corpora. However, at present the existing bilingual corpora are not sufficient (see Ota, 1998 for example); further investigation would be best carried out in a follow-up study that empirically probes for what these bilingual children do in all 8 conditions (as opposed to the 4 subset conditions we tested herein). We therefore leave this work for a future, separate follow-up. We also note that, while frequency in the relevant sense could potentially have some explanatory force for the results from Study 1 (bilingual vs. monolingual comparison), it could not contribute to an explanation for the results of Study 2 where access to English input is reduced to below 5% or so. As we believe the Study 2 data are the more interesting results and, crucially, constitute the novel focus of this paper on bilingual returnees, we leave the discussion of relative frequencies of genitive forms and the acquisition patterns for future work.

## Conclusion

There were two objectives in our study. First, we compared relative preferences for genitive forms and verb/argument orders in English between Japanese-English bilinguals and English monolinguals, to examine whether there are any effects of cross-linguistic influence in the former. The results showed that bilinguals differed from monolinguals only in the genitive conditions, specifically in those that required processing of semantic and/or pragmatic factors that are in conflict. These findings suggest that general processing difficulties in resolving such conflicts provide a better explanation for the observed behavior than does CLI from L1 to L2, as the Interface Hypothesis would predict. However, our results also show that not only the type of factors, but also their consistency plays a role in defining degrees of processing difficulties: therefore, it is necessary to go beyond simply contrasting “internal” and “external” interface conditions.

The second objective of our study was to investigate how severe change in continuous language input over time from the point of re-immersion in the L1 community affects returnee bilinguals’ L2 grammars. We set out to establish if there were any changes in the evaluation of genitive forms and of the verb/argument orders, and if so, whether the change(s) could be explained by increased CLI effects. Our results showed that there was no change in the preference for verb/argument orders; there was a change in the preference for genitive forms over time, but that it was restricted to a single condition, namely, the weak *s*-genitive condition. In order to account for the singling out of this condition, we proposed a principled explanation for why it is most susceptible to CLI. To be clear, the rather significant change in viewpoint, as compared to our original predictions, is a direct consequence of having learned from these data themselves. Combining the results from across the two studies, we believe the data come together to nicely show the dual effect of processing complexity and influence from dominant to non-dominant language, working in tandem to explain monolingual to bilingual differences as well as longitudinal changes within bilinguals over time.

## Data Availability Statement

The raw data supporting the conclusions of this article will be made available by the authors, without undue reservation.

## Ethics Statement

The studies involving human participants were reviewed and approved by The University of Edinburgh Linguistics and English Language Ethics Committee (protocol number 11–1516/5). Written informed consent to participate in this study was provided by the participants’ legal guardian/next of kin. Written informed consent was obtained from the minor(s)’ legal guardian/next of kin for the publication of any potentially identifiable images or data included in this article.

## Author Contributions

MK contributed to conceptualization, methodology, validation, carried out the formal analysis, investigation, data curation, funding acquisition, wrote the original draft, and visualized the data. CH contributed to conceptualization, methodology, wrote, reviewed and edited the manuscript, and supervised the data. AS contributed to conceptualization, wrote, reviewed and edited the manuscript, and supervised the data. JR wrote and reviewed and edited the manuscript. All authors contributed to the article and approved the submitted version.

## Conflict of Interest

The authors declare that the research was conducted in the absence of any commercial or financial relationships that could be construed as a potential conflict of interest.

## Footnotes

1 We should note here that the Interface Hypothesis has evolved away from a simple distinction between “internal” and “external” interfaces (see Sorace, 2016), toward a recognition that a range of different conditions can influence grammatical realization, some of which may be more taxing than others from a processing point of view.

2 In the initial study, we tested for an additional OSV condition such as *I like apples but oranges I hate* (OSV) vs. *I like apples but I hate oranges* (SOV). We have excluded this condition from the analysis since OSV verb/argument order in English is grammatical to the extent that it can be interpreted as involving an operation of “fronting/topicalization” that is felicitous only in a restricted range of discourse contexts (see e.g., Pullum, 1977). That is, we cannot be certain that the interpretation of OSV verb/argument order in our experiment does not involve more than one module of grammar (i.e., syntax), while the other three conditions in Figure 3 are clearly erroneous sentences and evaluating them does not require integration of semantic or pragmatic features. Eliminating this one condition from the analysis did not change the significance of the results in either Study 1 or Study 2.

3 Since age of L2 onset (AoO) and length of residence in the L2 environment (LoE) highly correlated (*r* = −0.85, *p* > 0.001), we were not able to include both variables in the model due to issues of multicollinearity. Therefore, we have decided to include AoO, as suggested by the reviewers, since our sample of bilingual returnee children included children with a wide range of AoO.
