# Supplementary material for: Cross-Linguistic Influence on L2 Before and After Extreme Reduction in Input: The Case of Japanese Returnee Children
Source: Front Psychol. 2020 Sep 18;11:560874. doi: 10.3389/fpsyg.2020.560874 (PMC7530843; doi:10.3389/fpsyg.2020.560874)
Supplement: Supplementary file 1 [file Data_Sheet_1.pdf]

Supplementary Online Materials Data Sheet 1

Table 1. Genitive items

Conditions			
[+animate] [+topical]		[-animate] [-topical]	
[+proto]	[-proto]	[+proto]	[-proto]
**strong *s*-genitive**	**weak *s*-genitive**	**weak *of*-genitive**	**strong *of*-genitive**
A bee stung a boy. The boy's nose/the nose of the boy was swollen for many days.	A new teacher came to our school. But nobody knew the teacher's name/the name of the teacher.	I banged my toe on a table's leg/the leg of a table and it hurts a lot.	It's annoying when people start laughing before a joke's end/ the end of a joke.
A man was feeding his pet bird. Suddenly, the man's bird/the bird of the man flew away.	A teacher made a joke about a boy. The teacher's joke/the joke of the teacher was very mean.	A girl is entering through a building's door/the door of a building.	A room's darkness/the darkness of a room makes people very anxious.
A little boy was looking at a baby because he wanted the baby's toy/the toy of a baby.	A woman was shouting on a train. The woman's voice/the voice of the woman was very loud.	I saw a toy I wanted in a shop's window/the window of a shop.	A story's beginning/ the beginning of a story is the most exciting part.
A girl was freezing outside. The girl's hand/the hand of the girl was very cold.	A man was drowning in a pool. A life guard saved the man's life/the life of the man.	My father fell down a house's chimney/the chimney of a house.	A leaf's colour/ the colour of a leaf changes as the season changes.

Table 2. Verb/argument order items

Conditions		
**SOV vs. SVO**	**SIODOV vs. SVIODO**	**SVDOIO vs. SVIODO**
*My dad newspapers everyday reads/ My dad reads newspapers every day.	*My uncle me a bag last week bought/ My uncle bought me a bag last week.	*Our teacher taught the alphabet us today/Our teacher taught us the alphabet today.
*My father those chocolates loves/ My father loves those chocolates.	*The customer the banker lots of money paid/ The customer paid the banker lots of money.	*The mother bought some crayons his son/The mother bought his son some crayons.
*The audience the concert very much enjoyed/ The audience enjoyed the concert very much.	*My best friend me a letter sent/ My best friend sent me a letter	*My friend told a scary story me last night/ My friend told me a scary story last night.
*The student an essay wrote/the student wrote an essay.	*She has never her sister a secret told/ She has never told her sister a secret	*Our teacher taught math us yesterday/ Our teacher taught us math yesterday.
