# Supplementary material for: Cross-Linguistic Influence on L2 Before and After Extreme Reduction in Input: The Case of Japanese Returnee Children
Source: Front Psychol. 2020 Sep 18;11:560874. doi: 10.3389/fpsyg.2020.560874 (PMC7530843; doi:10.3389/fpsyg.2020.560874)
Supplement: Supplementary file 2 [file Data_Sheet_2.pdf]

Supplementary Online Materials Data Sheet 2

Table 1. Estimated coefficients of the genitive model in Study 1: monolinguals vs. bilinguals

Fixed effects	Estimate	Standard error	*z*	*p*
Intercept	1.78	.34	5.20	<.001***
Monolingual	.49	.35	1.39	.16
Strong *of*-genitive	-2.34	.45	-5.19	<.001***
Weak *of*-genitive	-1.19	.44	-2.66	.007**
Weak *s*-genitive	-1.28	.45	-2.85	.004**
Monolingual : Strong *of*-genitive	-.76	.343	-1.75	.08
Monolingual : Weak *of*-genitive	-1.37	.43	-3.18	.001**
Monolingual : Weak *s*-genitive	.50	.44	1.11	.26

Note. **p* < .05; ***p* < .01; ****p* < .001

Table 2. Estimated coefficients of the verb/argument order model in Study 1: monolinguals vs. bilinguals

Fixed effects	Estimate	Standard error	*z*	*p*
Intercept	21.54	65.70	.32	.74
Monolingual	-16.23	65.69	-.24	.80
SIDV	-17.53	65.70	-.26	.79
SVDI	-19.24	65.70	-.29	.77
Monolingual : SIDV	17.49	65.70	.26	.79
Monolingual : SVDI	16.19	65.69	.24	.80

Note. **p* < .05; ***p* < .01; ****p* < .001

Table 3. Estimated coefficients of the genitive model in Study 2: bilinguals over time

Fixed effects	Estimate	Standard error	*z*	*p*
Intercept	1.81	.33	5.44	<.001***
Bilingual Round 2	-.39	.34	-1.16	.24
Strong of-genitive	-2.37	.43	-5.43	<.001***
Weak of-genitive	-1.22	.43	-2.81	004**
Weak s-genitive	-1.29	.43	-2.96	.003**
Bilingual Round 2: Strong of-genitive	.18	.42	.43	.53
Bilingual Round 2:: Weak of-genitive	.43	.42	1.02	.27
Bilingual Round 2:: Weak s-genitive	2.50	.54	4.62	<.001***

Note. **p* < .05; ***p* < .01; ****p* < .001

Table 4. Estimated coefficients of the verb/argument order model in Study 2: bilinguals over time

Fixed effects	Estimate	Standard error	*z*	*p*
Intercept	-.57	.26	-2.15	.03*
Bilingual Round 2	-.12	.26	-.46	.63
SIDV	1.13	.36	3.08	.002**
SVDI	2.29	.40	5.73	<.001***
Age	-.06	.06	-.97	.33
Bilingual Round 2 : SIDV	-.61	.35	-2.12	.09
Bilingual Round 2: SVDI	.74	.43	1.72	.08

Note. *$p < .05$; **$p < .01$; ***$p < .001$
